# Partners’ experiences of breastfeeding: a qualitative evaluation of a breastfeeding support intervention in Sweden

**DOI:** 10.1186/s13006-023-00609-6

**Published:** 2024-01-18

**Authors:** Ingrid Blixt, Ove Axelsson, Eva-Lotta Funkquist

**Affiliations:** 1 Department of Women’s and Children’s Health, Uppsala University, Uppsala, Sweden; 2 Centre for Clinical Research Sörmland, Uppsala University, Eskilstuna, Sweden

**Keywords:** Breastfeeding, Experiences, Infant, Intervention, Partner, Support, Qualitative methods

## Abstract

**Background:**

The World Health Organization states that women and their families need breastfeeding support from the healthcare system. However, knowledge about the most effective way to involve the partner in breastfeeding is lacking. A qualitative evaluation can provide insight and knowledge about the partner’s experiences towards a breastfeeding support intervention and thus contribute to how forthcoming breastfeeding support policies are designed. The aim of this study was to explore partners’ experiences regarding breastfeeding while participating in *The Breastfeeding Study*.

**Methods:**

An exploratory, longitudinal and qualitative design was used. This study was part of *The Breastfeeding Study*, which took place in Sweden. The intervention was performed in line with the Ten Steps to Successful Breastfeeding. Partners in the in the intervention group (IG) were part of a structured breastfeeding support programme. An individual breastfeeding plan was established in cooperation with the parents-to-be during pregnancy, and the plan was followed up at the child healthcare centre. A purposive sample was recruited from March to December 2021. Interviews and diary entries from IG (*n* = 8) and control group (CG) (*n* = 8) during pregnancy and 2 months after birth were analysed by content analysis, in accordance with the COREQ guidelines.

**Results:**

Partners’ experiences can be summarised under the main category of ‘Striving to be part of the family and important that the family’s everyday life was well-functioning’. IG partners experienced that both parents were involved and cooperated in the breastfeeding process and that guidance from healthcare professionals (HCPs) helped them to feel secure. CG partners experienced feeling excluded and not receiving support from HCPs.

**Conclusion:**

Both parents need to be targeted in breastfeeding support policies to meet the support needs. Midwives at antenatal care and child healthcare nurses at the child healthcare centre have important roles to play in providing structured breastfeeding support and a breastfeeding plan. Both IG and CG partners strived to become a part of the infant’s life and to make family life work. Midwives should involve both parents in a reflective dialogue on how the partner can be involved, apart from just feeding the infant.

**Trial registration:**

Retrospectively registered in ACTRN12623000648628.

**Supplementary Information:**

The online version contains supplementary material available at 10.1186/s13006-023-00609-6.

## Background

It is well known that breastfeeding has health benefits for the infant in both low- and high-income countries [1]. However, worldwide, only 44% of infants aged 0–6 months are exclusively breastfed [2]. The World Health Organization (WHO) states that women and their families need breastfeeding support from the healthcare system [2]. Previous research has, however, shown that the healthcare system does not meet these support needs [3].

Breastfeeding is the infant’s feeding norm, and its frequency has an important significance for infants’ and women’s health [1]. Since breastfeeding implies the best nutrition for infants, exclusive breastfeeding is recommended by the WHO for 6 months, followed by partial breastfeeding for 2 years or longer [2]. Most women are able to breastfeed [4], but the majority of women in high-income countries do not breastfeed exclusively for 6 months [1]; moreover, women often stop breastfeeding earlier than planned [5, 6]. In Sweden, exclusive breastfeeding among 2-month-old infants has declined from 80% in 2000 to 60% in 2020 [7]. The reason for this decline is mainly unknown.

Many factors affect whether women breastfeed [4]. For example, in high-income countries, women with higher education and higher income breastfeed longer [1]. Moreover, women with a partner with higher education breastfeed for longer periods [8]. Social and cultural factors, such as social trends, attitudes towards public breastfeeding, marketing of breastmilk substitutes, also affect breastfeeding [4]. Healthcare professionals (HCPs) can influence women’s decision to breastfeed by providing good support [4]. The Infant-Friendly Hospital Initiative and the Ten Steps to Successful Breastfeeding (Ten Steps) to protect, promote and support breastfeeding are recommended as the best standard for support [9]. Step three in the Ten Steps states that the HCP should involve partners in the dialogue about the importance and management of breastfeeding [10].

A previous report concerning high-income countries shows that the partner’s attitude towards breastfeeding can influence women’s intention to breastfeed exclusively [11]. Furthermore, a study from Sweden, Ireland and Australia finds that positive attitudes and support from the partner are essential for women to be able to maintain breastfeeding for 6 months. Women value a partner who shares housework, cares for her and does not pressure her to give commercial milk formula to the infant [12]. A study from Canada shows a positive influence on breastfeeding duration when the woman perceives that the partner provides sensitive and responsive support based on her needs [13]. On the other hand, this study also reports that when the partner is more knowledgeable about breastfeeding, provides practical support and is more involved in the infant’s care, a negative influence is observed on women’s intention to breastfeed and breastfeeding duration [13].

A review of partners’ experiences and perspectives of breastfeeding reports that the partner’s primary goal is to bond with the infant. Partners perceive that breastfeeding can be a barrier for them to bond, and they often feel left out during the breastfeeding period. The partners describe feeling excluded from breastfeeding decisions [14]. Another study found that partners are unsure of their role in decisions about breastfeeding and how to provide breastfeeding support [15]. Partners describe that they provide support by caring for the mother, changing the infant’s diaper and taking care of older siblings. They appreciated being able to provide practical support by bottle-feeding the infant with expressed breastmilk or formula. They also want to have information from the HCP on breastfeeding problems, on how to support the mother, on a suitable diet for breastfeeding women, on how and when to wean the infant and how to manage their own feelings of jealousy [14].

A systematic review [16] and a meta-analysis of breastfeeding interventions [17] report that if the partner is involved in the intervention, it can increase breastfeeding initiation, exclusivity and duration. Effective interventions during pregnancy and after birth are associated with face-to-face support by trained HCPs [16]. In contrast, another systematic review found that if the partner is involved, it can decrease breastfeeding exclusivity and that some women cease breastfeeding due to advice from the partner [18]. In summary, knowledge about the most effective way to involve the partner in breastfeeding interventions is lacking, and little attention has been paid to partners’ perceptions and experiences of breastfeeding interventions [16]. A qualitative evaluation ought to provide insight and knowledge about partners’ experiences towards a breastfeeding support intervention and thus contribute to how forthcoming breastfeeding support policies are designed.

## Methods

### Aim

The aim of this study was to explore partners’ experiences regarding breastfeeding while participating in *The Breastfeeding Study*.

### Design

This study has an exploratory, longitudinal and qualitative design and reports data from interviews and diary entries collected at two time points: during pregnancy and 2 months after the infant’s birth. Data were analysed by content analysis, as described by Elo and Kyngäs [19]. The study includes partners who participated in a breastfeeding intervention study [20], where couples were offered breastfeeding support based on the Ten Steps [10]. Data for this study were collected from March to December 2021.

### Sample/participants

#### Setting/breastfeeding support policies

The study setting and healthcare system in the region have previously been described in detail [20].

#### The intervention

During the years 2020–2022, partners were not allowed to attend the visits to the antenatal care due to the COVID-19 pandemic. However, they were allowed to attend the visits to the child healthcare centre.

The breastfeeding support programme in the intervention group (IG) is described below and in Additional file 1.

1. Antenatal care:
  - Pregnant women received structured breastfeeding counselling at the antenatal clinic during normal visits at pregnancy weeks 28, 32 and 38 (5–10 min). The midwife encouraged the woman to share and fill in the breastfeeding plan together with her partner. The parents-to-be also watched short online breastfeeding lectures before the visit in pregnancy week 32. The midwife went through the breastfeeding plan with the woman at the visit in pregnancy week 32. The plan included questions about the mother’s intentions, the couple’s experiences and expectations, as well as what kind of breastfeeding support they wanted from their family and HCPs.
  - An individual breastfeeding plan was established in cooperation with the parents-to-be. The plan included:
    1. Self-studies during pregnancy,
    2. QR-codes for four short online breastfeeding lectures, and
    3. QR-codes for two leaflets.
  - The midwife followed up on the breastfeeding plan during the normal visit 8–12 weeks postpartum.
2. Child healthcare centre:
  - Parents received structured breastfeeding counselling during the normal visits at 2 and 6 weeks, as well as 3 and 5 months postpartum (5–10 min).
  - The child healthcare nurse (CHCN) followed up on the breastfeeding plan at each visit.

#### Standard care in the control group (CG)

The midwife at the antenatal care talked about breastfeeding, and women received a paper leaflet during the visit in pregnancy week 28. The midwife did not encourage the women to share the standard leaflet with their partners.

#### Sample

The 16 partners (8 IG and 8 CG), who consented to participate, were partners to the women described in the breastfeeding intervention study. The women were recruited using maximum variation purposive sampling based on education, age and parity [21]. There were no dropouts. The inclusion criteria were that all women were healthy and, at pregnancy week 24, planned to initiate breastfeeding after birth. Another criterion was that the partner could communicate in Swedish. Characteristics of the participating partners are displayed in Table 1. Five out of seven infants in the IG and four out of eight in the CG were exclusively breastfed or received only human milk at the age of 2 months (Table 2).

**Table 1:** Characteristics of partners and mothers

	Intervention	Control
	*n* = 8^a^	*n* = 8
**Partners**		
Age, mean (range)^a^	35 (29–36)	32 (29–45)
University education, n (%)^a^	2 (25.0)	4 (50.0)
Household income > 40,000 SEK/4000 EUR per month n (%)^a^	6 (85.7)	5 (62.5)
Male sex	8 (100.0)	7 (87.5)
Born in Sweden^a^	5 (71.4)	7 (87.5)
Interviews during pregnancy, n (%)	4 (50.0)	3 (37.5)
Diaries during pregnancy, n (%)	0 (0)	1 (12.5)
Interviews 2 months postpartum, n (%)	5 (62.5)	3 (37.5)
Diaries 2 months postpartum, n (%)	1 (12.5)	3 (37.5)
**Mothers**		
Previous experience of breastfeeding n (%)	4 (50.0)	4 (50.0)
Plan at gestational week 24 for duration of exclusive breastfeeding^a^		
*No plan*	2 (25.0)	0 (0.0)
*4 to 5 months*	1 (12.5)	5 (62.5)
*6 months*	4 (50.0)	3 (37.5)

^a^Data from one mother is missing

**Table 2:** Feeding during the first 2 months

	Intervention	Control
	*n* = 8^a^	*n* = 8
Exclusive breastfeeding, n (%)	3 (43.0)	4 (57.1)
Breastfeeding/ Human milk, n (%)	2 (29.0)	0 (0.0)
Breastfeeding/ Human milk and Formula, n (%)	2 (29.0)	4 (50.0)

^a^Data from one mother is missing

### Data collection

Since recruitment of partners at antenatal care was impossible due to the COVID-19 pandemic, they were contacted by a member of the research team (IB) by telephone and invited to participate. After consent was obtained, partners could choose whether they wanted to be contacted for an initial telephone interview at a time that suited them or to complete diary entries with the same questions via mail (Table 1). Partners who chose an interview were informed that the interviewer was a female midwife (IB) with experience in providing support to breastfeeding families, as well as in performing qualitative telephone interviews. Submission of an online diary was considered as consent to participate. The pregnant women also provided their written consent, and baseline data were collected (Table 1).

The telephone interviews started by providing information about the purpose of the study and obtaining consent to record the interview. Data were collected using a semi-structured interview guide. The interview guides and diary questions focused on partners’ experiences of breastfeeding, with questions such as, ‘*Could you please explain if you have experienced any advantages of breastfeeding*’. ‘*Could you please explain if you have experienced any disadvantages of breastfeeding*’. Probing questions were used, for example, ‘*Could you please tell me more about that*’. The woman was not present during the interview, which had a mean duration of 26 min; following the interview, field notes were taken. The questions were based on a literature review and experiences within the research group. One pilot interview was conducted in the IG and one in the CG to test the technique and the appropriateness of the questions. No changes were made, so the pilot interviews were included in the study.

### Data analysis

**Fig. 1:**
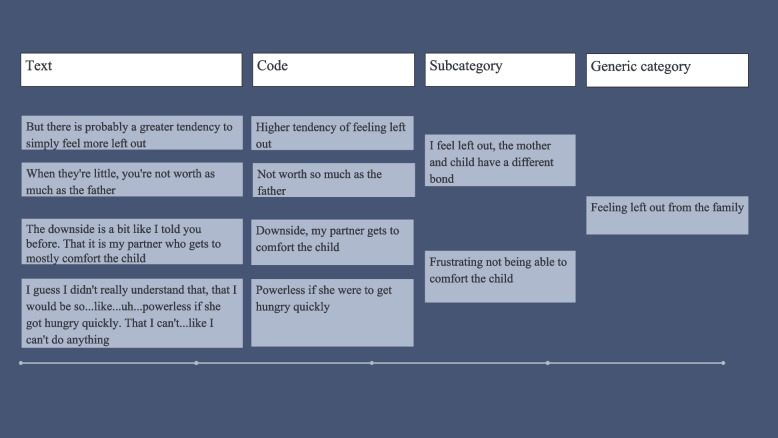
Example of the initial coding process

**Fig. 2:**
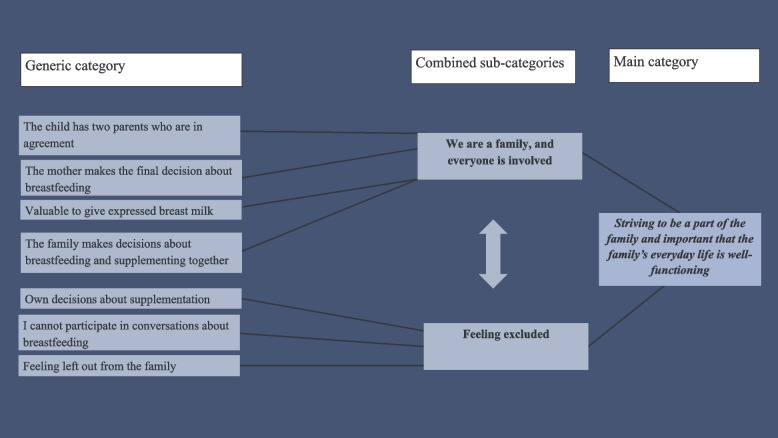
Example of the analysis process for the generic category, combined sub-categories and main category

**Fig. 3:**
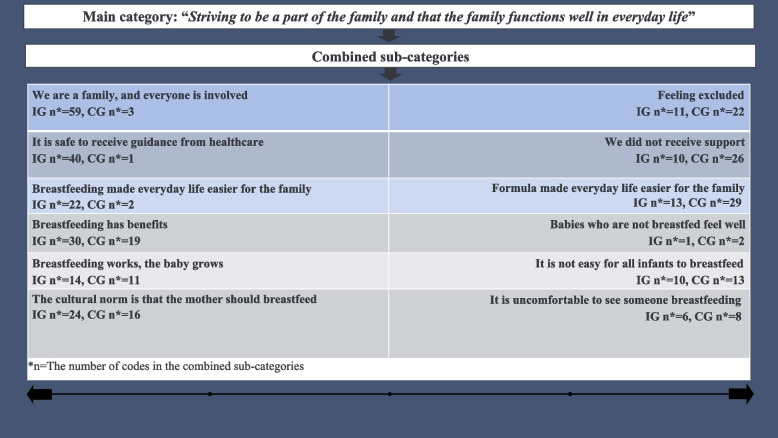
Main category and combined sub-categories. *n = The number of codes in the combined sub-categories

A content analysis with an inductive approach was chosen to determine partners’ experiences regarding breastfeeding [19]. During the data collection period, the researchers discussed the field notes, sharing their reflections and initial insights. In the first step, interviews were transcribed, and the transcripts from all interviews and the text from the diaries were read and re-read until it became familiar and had a sense of “meaning” [19]. In step two, authors (IB and EF) coded the data separately. Narratives related to the aim were highlighted [19]. In step three, the contents of the different narrative units were described using initial codes [19]. In step four, a discussion within the research group led to an agreement on coding (Fig. 1). Codes were merged into preliminary sub-categories. In step five, the sub-categories were merged into generic categories and further into combined sub-categories based on similarities and differences in the content (Fig. 2) [19]. The research team discussed the coding until an agreement was reached. In step six, all researchers participated in the abstraction process, which resulted in the main category: *Striving to be part of the family and important that the family’s everyday life was well-functioning* (Fig. 3).

In the last step, the number of codes in the combined sub-categories was quantified to provide insights into similarities and differences in the IG and CG, using summary content analysis [22], (Fig. 3). A professional translator translated the quotes into English.

### Rigour

The research team used credibility and dependability to enhance the trustworthiness of the study. To improve credibility, the researchers combined data from the semi-structured interviews and diaries during pregnancy and after birth [21], which gave access to partners’ experiences of breastfeeding over time. To improve credibility during the analysis process, the researchers engaged in a continuous movement back and forth between the codes and sub-categories and between the sub-categories and generic categories as well as between the generic categories and combined sub-categories during the analysis. The research group discussed the coding until an agreement was reached [23]. The background and previous experiences within the research team are important aspects of qualitative research [21, 23, 24]. Our team consists of two female midwives, one female CHCN and a male physician. Two of the authors (IB and EF) have extensive professional experience in supporting expectant couples and families with breastfeeding guidance. They had positive experiences of breastfeeding and partner support themselves. To reduce the risk of bias an expert in content analysis, who had no prior knowledge about the intervention, took part in the analysis. The team was reflective during the data collection and analysis according to their prior understanding and own experience of breastfeeding. To improve dependability, the research team developed the semi-structured interview guide [23]. The study follows the Consolidated Criteria for Reporting Qualitative Research (COREQ) for interviews and focus groups [24].

## Results

### Striving to be part of the family and important that the family’s everyday life was well-functioning

The main category, ‘*Striving to be part of the family and important that the family’s everyday life was well-functioning*’, shows that partners in both groups felt it was important to be part of the family and that the family’s everyday life was well-functioning (Fig. 3).

The main category is described through six combined sub-categories, where each sub-category describes a process with opposite poles of partners’ experiences regarding breastfeeding (Fig. 3). IG partners experienced that both parents were involved and cooperated in the breastfeeding process. Guidance from HCPs helped them to feel secure, and sharing the feedings with breastmilk made everyday life easier for the family. CG partners felt excluded and did not feel they received support from HCPs. Sharing the feedings with formula made everyday life easier for the family. Both groups experienced benefits of breastfeeding during the breastfeeding process. Their experience was influenced by the cultural norm.

### We are a family, and everyone is involved – feeling excluded

#### We are a family, and everyone is involved

The IG partners felt both parents were important and that they cooperated in the family. They were pleased when the pregnant women shared the breastfeeding information she received from the midwife. During pregnancy, IG parents talked a lot about breastfeeding and the woman’s previous breastfeeding experiences. IG parents filled in the breastfeeding plan during pregnancy, watched the movies and read the leaflets about breastfeeding: *‘We looked through the brochures; we read them, and we watched the films together and we talked about it’*. (Interview IG, Informant 14). After birth, IG partners were often present when the infant was breastfed and viewed it as an enjoyable experience. They stated that both parents were important and that the infant could be satisfied by both parents. IG partners created their own relationship with the infant and had, for example, skin-to-skin contact with the infant between feeds. They bathed the infant or walked with the infant in the stroller without the participation of the mother: *‘I have taken him out in the pram myself and sit with him a lot. It has been great fun. I have been able to be very involved. There is no difference between us’.* (Interview IG, Informant 12)*.*

They also described being pleased with their communication and sharing household chores after birth:

*‘We have been able to...communicate in a good way...you talk so that you are on the same page all the time. But also that you share the practical because it has to work as well. So we have to pump. But that’s not a disadvantage; it’s just that you have to be a little organised’.* (Interview IG, Informant 7).

IG partners valued when the women expressed breastmilk, so that they could feed the infant. They wanted the mother to breastfeed as much as possible but stated that it was up to her to decide whether to breastfeed. Partners in both groups stated that decisions about continued exclusive breastfeeding or introducing bottle feeding with formula were made by both parents.

#### Feel excluded

CG partners stated they made their own decisions to feed the infant with formula if the mother was not available and the infant cried:

*‘She does not like it because she would much rather continue breastfeeding...if she wants to go out for a bike ride in the evening…If he screams. And if he’s hungry and [name] isn’t here, he gets to eat from the bottle’.* (Interview CG, Informant 11).

First-time CG partners stated they felt left out during pregnancy, since their lack of knowledge was a barrier to talking about breastfeeding with the pregnant mother. They had confidence in the woman’s knowledge: *‘I’m leaning on my partner; what food will we need to buy in the beginning, or is it just breastfeeding’.* (Interview CG, Informant 16).

During pregnancy, family and friends of partners in both groups warned them they might feel excluded after birth, stating it was only the mother who mattered to the infants for a long time. Partners in both groups said that a disadvantage with breastfeeding was that the mother could form a unique bond with the infant, making them feel left out. After birth, both groups articulated feelings of being frustrated, incapable, worthless and jealous: *‘It’s frustrating. I’m standing there with a screaming bundle; it doesn’t matter what I do…it’s the mother’s breast that matters’.* (Interview CG, Informant 2).

Some partners wished they could breastfeed. Partners with older children, in both groups, stated they knew this was a transient period.

### It is safe to receive guidance from healthcare – we did not receive support

#### It is safe to receive guidance from healthcare

IG partners stated that both parents had received fact-based information about breastfeeding during pregnancy. They had gained knowledge through movies and leaflets: *‘I didn’t know why you breastfeed. I learned about the benefits of breastfeeding. That there is so much positive that comes with breastfeeding for the child and for the mother’.* (Interview IG, Informant 9).

After birth, IG partners uttered that HCPs respected the breastfeeding plan. They were pleased because HCPs involved both parents in the dialogue on breastfeeding. The HCP asked how the parents experienced breastfeeding, how it worked and about support needs. CHCNs were perceived as non-judgmental and as guides for families to find ways to breastfeed. The CHCN asked about breastfeeding at every visit:

*‘We are involved in the discussion there, both me and [name]. We talk very openly there. All three of us. As [name] breastfeeds, so...there has been a standing discussion at every visit, how it works. Every single visit’.* (Interview IG Informant 12).

The CHCN assured IG parents that the infant was satisfied and growing because of their care and helped parents to solve breastfeeding problems. IG partners trusted the advice from the CHCN, which implied that they did not read much about breastfeeding. IG partners perceived it as safe to receive guidance from the CHCNs: *‘I think it feels safe to have that guidance, and she has been positive about breastfeeding, our nurse; it has felt good’.* (Interview IG, Informant 14).

#### We did not receive support

CG partners explained that they received no, or very little, information about breastfeeding. They were pleased when the CHCN confirmed that formula was a good alternative to breastfeeding during the first months after birth: *‘I am very happy with our nurse, and we have largely the same opinion; we changed the evening meal at around 8 pm to supplementing with formula’.* (Diary CG, Informant 6).

Partners in both groups stated that some HCPs had more positive attitudes towards formula feeding than towards breastfeeding.

First time CG partners were also disappointed because they had not been allowed to visit “the expert” (midwife) during pregnancy. This made them feel left out and made it more difficult for them to find their role. Such feelings caused irritation between parents:

*‘It’s sad that, as a father, especially now that it’s Covid, I can’t go to the routine check-ups; I’m not the one who breastfeeds. The mother gets everything. While the father gets a bit like this “uh, you have to grasp at a corner there”...we have managed to get annoyed with each other’.* (Interview CG, Informant 15).

### Breastfeeding made everyday life easier for the family –formula made everyday life easier for the family

#### Breastfeeding made everyday life easier for the family

IG partners said it was convenient when both parents could share feedings, since it simplified everyday life. They preferred bottle feeding with breastmilk in the first place, but it could be topped up with formula when the breast milk was not enough:

*‘Practically - If there’s a moment when it’s just me at home. We think it’s important that he gets as much breast milk as possible and it’s good that...she has time to pump and that there is...in a bottle’.* (Interview IG, Informant 7).

Partners in both groups stated that breastfeeding made everyday life easier. For example, breastfeeding was practical and convenient because food was always available, heated and ready. Partners with earlier experiences of formula feeding perceived that breastfeeding helped parents to sleep better and that co-sleeping with the breastfed infant contributed to improved sleep:

*‘I have two children with another partner. There, it was not possible to breastfeed; it was supplementing with formula. I am used to getting up at night and doing it. So, I think it worked great. He wakes up and is nursed and goes back to sleep’.* (Interview IG, informant 9).

#### Formula made everyday life easier for the family

After birth, CG partners with older infants stated they became exhausted because they took responsibility for older siblings, housework, job, finances and housing. They also said they had no one to talk to about their experiences. They said that it was convenient when both parents could share feedings but by bottle feeding with formula, since it simplified everyday life: *‘Supplementing with formula is, of course, a welcome option, to be able to share the feedings’.* (Diary CG, Informant 6).

Both groups stated it was a disadvantage that the mother did not have time for anything else except for breastfeeding and that she was unable to do anything without the infant. They saw it as important to relieve the mother during the breastfeeding period and wished they could help her more. During pregnancy, partners worried about whether they would be strong enough to both work and take responsibility for everything, apart from feeding the infant. For example, they were unsure if they would have enough time to eat or sleep.

### Breastfeeding has benefits – infants who are not breastfed feel well

#### Breastfeeding has benefits

IG partners talked about specific benefits and about breastfeeding being less stressful for the mother and infant and that breastfeeding promotes the infant’s attachment:

*‘As much antibodies as possible. Before, I probably thought that it was just a matter of supplementing with formula. But it doesn’t feel like that anymore, considering that we have come to understand that breast milk contains a lot more than just energy’.* (Interview IG, Informant 9).

Both groups said that breastfeeding provides emotional closeness between the woman and infant and stated that breastfeeding can comfort the infant.

#### Infants who are not breastfed feel well

Other partners in both groups described that breastfeeding and formula were equally good: *‘There are children who have been separated from their mothers at birth and have grown up eating and drinking and are doing just fine’.* (Interview CG, Informant 16).

### Breastfeeding works, the infant grows – not all infants find it easy to breastfeed

#### Breastfeeding works, the infant grows

IG partners described being happy when breastfeeding was working 2 months after birth and that the infant received enough breastmilk and grew: *‘I’m happy that my wife wants to breastfeed and that it works’.* (Diary IG, Informant 5). One CG partner stated he was happy that his wife did not give up when problems arose, because now breastfeeding finally worked. Other CG partners stated they did not have a breastfeeding plan, but the infant continued to breastfeed because it worked so well.

#### Not all infants find it easy to breastfeed

During pregnancy, partners in both groups had heard from family and friends that breastfeeding can work, but it could also be very problematic. They had been advised to buy formula before birth. Both groups also experienced problems during the first period after birth, such as painful breastfeeding, the infant not gaining weight or the mother feeling that the infant did not want to breastfeed.

### The cultural norm is that women should breastfeed – it is uncomfortable to see someone breastfeeding

#### The cultural norm is that women should breastfeed

Partners from both groups believed that breastfeeding is the natural way to feed the infant. They disliked the breastfeeding norm and felt that it put pressure on women who did not want to or could not breastfeed. For example, partners stated women did not want to breastfeed because breasts are sexual objects for their partner. Both groups felt that women could experience shame if they were unable to breastfeed and that women put pressure on each other on social media. Partners also mentioned that information early in pregnancy had influenced their understanding about breastfeeding:

*‘The perception we had that we should breastfeed fifty/fifty comes from that book where they say “yes, but it’s clear that only breastfeeding is the best”, but because we have clean water in Sweden, you can feel safe with buying and using the others’.* (Interview IG, Informant 7).

#### It is uncomfortable to see someone breastfeeding

During pregnancy, partners in both groups stated breastfeeding was normal; however, they were unsure if it was acceptable to breastfeed in public:

*‘It is completely normal to breastfeed. It doesn’t matter where...she is. Because there is nothing strange about it. You know, people say like this, “it’s dirty, it’s going to be naked.” It’s this and that’.* (Interview CG, Informant 15).

## Discussion

The purpose of this qualitative study was to explore partners’ experiences regarding breastfeeding. The main findings were that partners strived to be part of the family and felt it was important that the family’s everyday life was well-functioning. IG partners thought that both parents were involved and cooperated in the breastfeeding process. Guidance from HCPs helped them to feel secure. Sharing feedings with breastmilk made everyday life easier for the family. CG partners felt excluded and did not feel they received support from HCPs. Sharing feedings with formula made everyday life easier for the family. Partners from both groups were aware of the benefits of breastfeeding during the breastfeeding process.

### Everyone is involved

IG partners believed both parents were important and that they cooperated in the breastfeeding process. The midwife provided the parents-to-be with a breastfeeding plan that included tools to start talking about expectations, experiences and needs during pregnancy. Co-parenting has been described as a way for parents to work together. The parents supported each other by affirmation of the other parent’s competence [25]. IG partners felt happy that both parents were important and that they had created their own relationship with the infant. For example, they described having skin-to-skin contact with the infant, bathing the infant or walking with the infant in the stroller, which may have strengthened their self-confidence as parents. Our results indicate that the intervention evoked partners’ experiences of being involved and increased their interaction with their infant. Furthermore, the results from our study support the findings from a Canadian study on co-parenting breastfeeding support, in which the partners perceived that information on how to be involved with their infant was very helpful [26].

An unexpected finding was that IG partners were pleased because mothers provided support by sharing household chores, so that the partner could also feed the infant with expressed breastmilk. This is congruent with the findings from a previous breastfeeding intervention study [26]. Cultural norms affected both IG and CG partners, and in Sweden, partners often want to be an equal parent [27]. Partners may perceive feeding the infant as being an equal, instead of spending time on household chores, which they see as tiring [28]. There are advantages in using expressed breastmilk, instead of formula. However, a study from the US suggests that women often stop breastfeeding earlier than planned because they perceive that pumping breastmilk is not worth the effort [5]. Partners in both groups believed that bottle-feeding enhances the bonding process with the infant, which is a finding previously reported by Baldwin et al. [29] and Sihota et al. [14]. Perhaps this notion includes the belief that bottle-feeding facilitates the infant’s attachment to the bottle-feeding partner. Such a conception is a simplification and a myth, because attachment is considerably more complicated. To create a secure attachment to the infant, the parent needs to be sensitive and have an appropriate responsiveness to the infant’s needs [30]. One must also keep in mind that when it comes to attachment and bonding, it is the infant, not the partner, who should feel validated. Feinberg [25] states that co-parenting does not imply that parents’ roles are or should be equal in all areas of responsibility. A study by Abbass-Dick and Dennis [31] indicates that, in an effective co-parenting relationship, parents set breastfeeding goals together.

In contrast to IG, CG partners stated they made their own decisions to feed the infant with formula if the mother was not available and the infant cried. In fact, one parent could undermine the other by not upholding the decision not to give formula. Our results show that both groups described feelings of being frustrated, incapable and worthless during the breastfeeding period. These views are similar to a previous review [14], where partners stated that their inability to feed their infant led to low-self-efficacy as a parent. Furthermore, our results show that first-time CG partners felt their lack of knowledge was a barrier to discussing breastfeeding in the breastfeeding process. It has previously been claimed that first-time partners often feel unprepared for how to support women during breastfeeding and that this makes them feel helpless [29].

### Guidance from healthcare

IG partners stated it was helpful to receive fact-based knowledge about breastfeeding during pregnancy. According to the Ten steps, HCPs should provide expectant parents with knowledge about the importance and management of breastfeeding [10]. A breastfeeding support intervention from Canada has shown that partners who received breastfeeding knowledge postpartum perceived it as helpful [26]. In our study, CHCNs were highlighted as important persons by IG partners because they involved both parents in a safety-creating dialogue. Thus, it appears that structured breastfeeding counselling from well-trained HCPs can affect partners’ well-being [16].

CG partners, who did not receive structured breastfeeding counselling, claimed they received no, or very little, information. These findings confirm previous findings in showing that midwives focus on preparing pregnant women for birth [3]. In fact, partners often reported they learned about breastfeeding and formula feeding from the Internet [14]. This evaluation shows that CG partners were pleased when CHCNs confirmed that the family could introduce formula. One could assume that partners believed the HCPs advice was based on evidence. Furthermore, CG partners in this study were disappointed because they were not allowed to participate in the visits during pregnancy. However, neither the IG nor the CG was allowed to participate in the visits due to the COVID-19 pandemic. CG partners stated that feeling excluded caused irritation between the parents, and such feelings are risk factors for postpartum depression [32].

### Everyday life

IG partners preferred to give the infant expressed breastmilk, instead of formula. In contrast, CG partners claimed that sharing feedings with formula simplified everyday life for the family. A previous study [14] found that partners perceive formula as being more convenient than breastfeeding. A study from Sweden has shown that adolescents often have positive beliefs and attitudes towards shared parenting [33]. Swedish parenting books often present bottle feeding as more gender equal than breastfeeding [34]. Parents often report that they trust feeding advice from books written by authors regarded as experts [35]. Both IG and CG partners reported that their positive attitudes towards shared feeding have been influenced by parenting books, and they had read that formula is almost equivalent to breastfeeding. Several IG and CG partners had a low level of education, which is often associated with having trust in the information provided by the commercial milk formula industry [35]. In line with the results from a study involving British partners with low education [36], both IG and CG partners were concerned that the mother would receive negative comments if they breastfeed in public. In this evaluation, both IG and CG partners thought it was important to relieve the woman during the breastfeeding period. However, they were also worried about not being able to cope with the responsibility for everything, except feeding the infant. Furthermore, CG partners with older children also felt they became exhausted because they took responsibility for everything but had no one to talk to. Parents with low education [1, 8] may have role models who prefer formula feeding in their social network [12]. If the families’ network has negative attitudes towards their feeding choices, it can lead parents to stop seeking support, causing them to hide the fact that the woman is breastfeeding [12, 35]. Parents often choose the feeding method that reduces stress in their family [35], and infants of parents with low education often breastfeed for shorter periods [1, 8]. If women perceive breastfeeding as the cultural norm, this will influence breastfeeding positively [12]; therefore, it is important that the society has positive attitudes towards breastfeeding. In a previous report, partners identified HCPs as important persons if they needed support [3]. However, families with a low educational background do not participate in parental groups to a great extent [37]. Therefore, breastfeeding support policies should encourage both first-time partners and partners with older children to attend the antenatal visits and thereby take part in the structured breastfeeding counselling provided by midwives. Such a policy should facilitate partners to share experiences and receive support already during pregnancy.

### Health aspects

IG partners talked about the specific benefits of breastfeeding; for example, they mentioned it strengthened the infant’s immune system. We know that partners often lack knowledge about the benefits of breastfeeding [14]. Young males often have more negative breastfeeding norms than females [33], which underlines the importance of providing partners with knowledge about the health benefits of breastfeeding. IG and CG partners described that an advantage with breastfeeding was that women could form a close bond with the infant. These results are congruent with another study which reported that Swedish women perceive that breastfeeding could facilitate a feeling of emotional closeness with the infant, which they saw as a motivation for maintaining breastfeeding [12]. In contrast, other IG and CG partners stated formula was as good as breastfeeding, which highlights the importance of identifying partners with negative attitudes towards breastfeeding and providing information about the benefits of breastfeeding to them.

### Implications for research and practice

Our results provide HCPs with new knowledge on partners’ experiences of the importance of being part of the infant’s life and making family life work. The breastfeeding plan can be used to provide partners with knowledge and thus, facilitate parents-to-be to talk with each other about their support needs. This may be especially important for families where partners have difficulties attending the antenatal visits and taking part in the parental education, which often is the case for socio-economically disadvantaged parents [37]. CHCNs have an important role in providing structured breastfeeding support and to follow-up on parents’ support needs.

HCPs need to be aware of partners’ need to form a close relationship by sharing the feedings. Therefore, it is important that midwives involve both parents-to-be in a reflective dialogue, where they explain how breastfeeding works and how the partner can be involved, apart from bottle-feeding the infant. Currently, we do not know if sharing the feedings with breastmilk in bottles has an influence on exclusive breastfeeding.

### Limitations and strengths

Effective breastfeeding interventions that involve the partner have been associated with face-to-face support from trained HCPs [16]. However, due to the COVID-19 pandemic, IG and CG partners were not allowed at the antenatal care clinics. This may have influenced our results. Our interviews were not performed face-to-face, which might have limited the depth of the study [38]. However, partners may feel more relaxed about sharing their experiences in a telephone interview or by writing in a diary. Moreover, the partners could participate without the need to travel [38]. Our study sample was small but large enough to provide a variety of experiences from partners [23].

The design of the intervention may have affected the engagement of participants having a low educational level. This is a group that rarely participates in parenting support, but the intervention resulted in partner participation. A study on a co-parenting breastfeeding support intervention recommends that information should target both parents and be delivered in a variety of modes [26].

This study was conducted in one region in Sweden; thus, the results cannot be generalised across different international or cultural contexts. Using maximum variation purposive sampling [21], based on education, age and parity may have influenced the results. Several of the partners in both groups in this study had a low educational level, for example.

## Conclusions

Partners who received the intervention experienced that both parents were important, were involved and cooperated in the breastfeeding process. Such results strengthen partners’ self-confidence as parents. Midwives and CHCNs have an important role to play by providing structured breastfeeding support and a breastfeeding plan with self-studies. Partners should be targeted in breastfeeding support policies to meet their need for support. Such a policy can decrease their feelings of being left out.

Both IG and CG partners strived to become a part of the infant’s life and to make family life work. Midwives at antenatal care should involve both parents-to-be in a reflective dialogue to explain how breastfeeding works and how the partner could be involved, apart from feeding the infant.

### Supplementary Information

**Additional file 1.** Supporting information.

## Data Availability

The datasets used and/or analysed during the current study are available from the corresponding author upon reasonable request.

## Abbreviations

CHCN: Child healthcare nurse
CG: Control group
HCP: Healthcare professional
IG: Intervention group
Ten Steps: Ten Steps to Successful Breastfeeding
WHO: The World Health Organization
