# Supplementary material for: Partners’ experiences of breastfeeding: a qualitative evaluation of a breastfeeding support intervention in Sweden
Source: Int Breastfeed J. 2024 Jan 18;19:6. doi: 10.1186/s13006-023-00609-6 (PMC10797796; doi:10.1186/s13006-023-00609-6)

The evidence-based picture and conversation material for mothers

TALKING ABOUT SKIN-TO-SKIN AND BREASTFEEDING IN ANTENAL CARE

The evidence-based picture and conversation material used by midwives during antenatal care: They used empathetic, reflective listening with open-ended questions, reflected and summarised. Healthcare professionals explore what the breastfeeding family already knows about breastfeeding, ask for permission to provide information and offer information.

Additional file 1. Supporting information

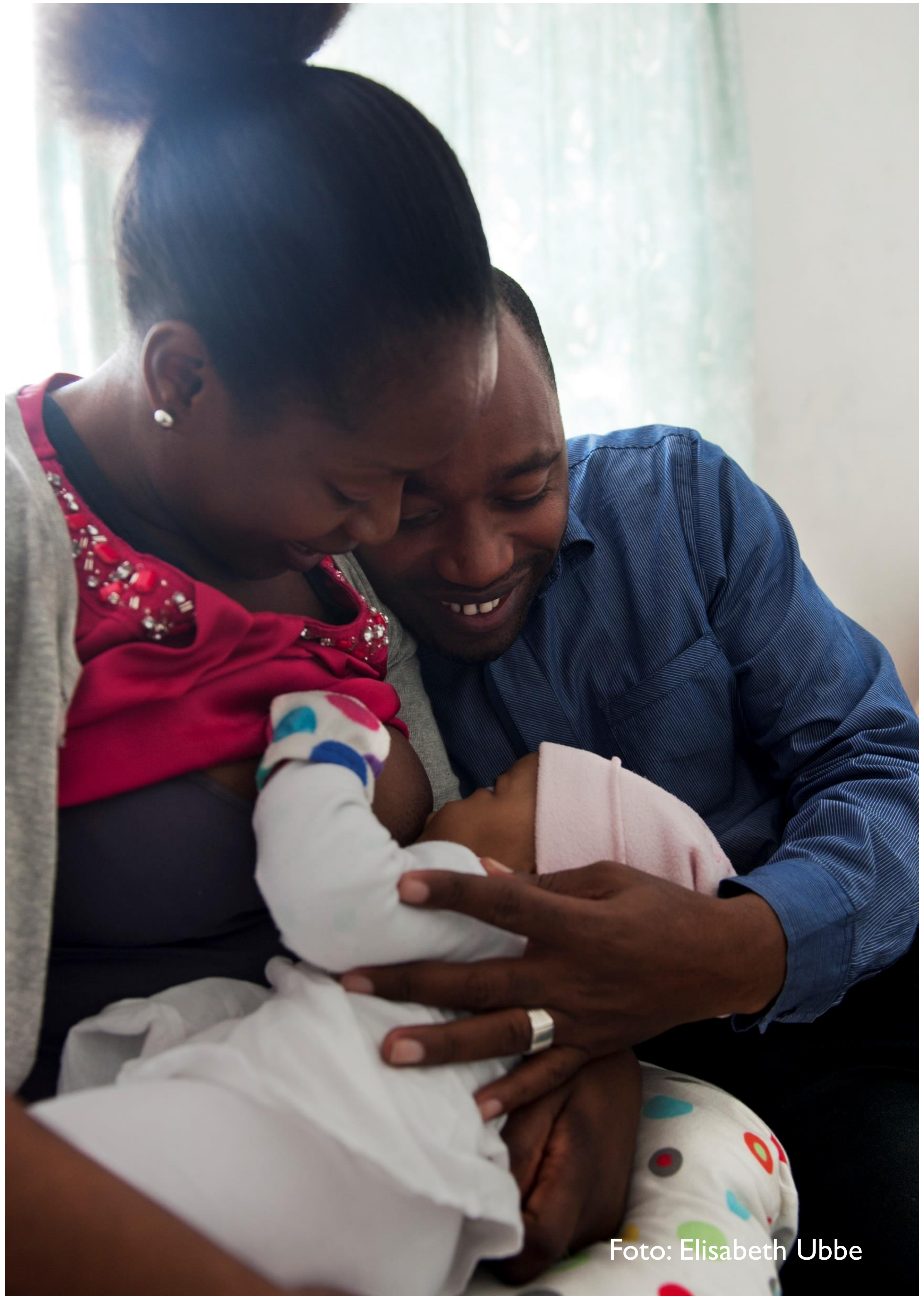

At the antenatal care visit during pregnancy week 28

- Health benefits for mother and baby
- Parents receive the breastfeeding plan, and the midwife fills in where to get help with breastfeeding during the breastfeeding period

Parents’ self-studies

- The midwife encourages parents to read and fill in the breastfeeding plan together and to watch the short online breastfeeding lectures for parents in the breastfeeding plan before the visit in pregnancy week 32

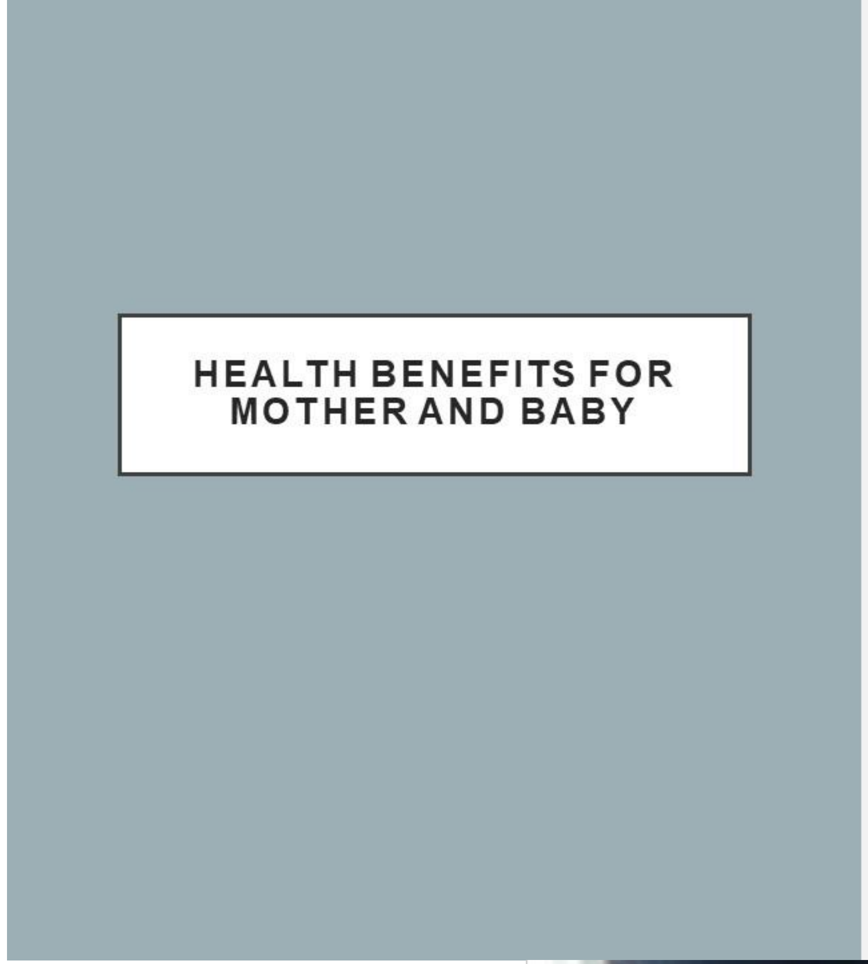

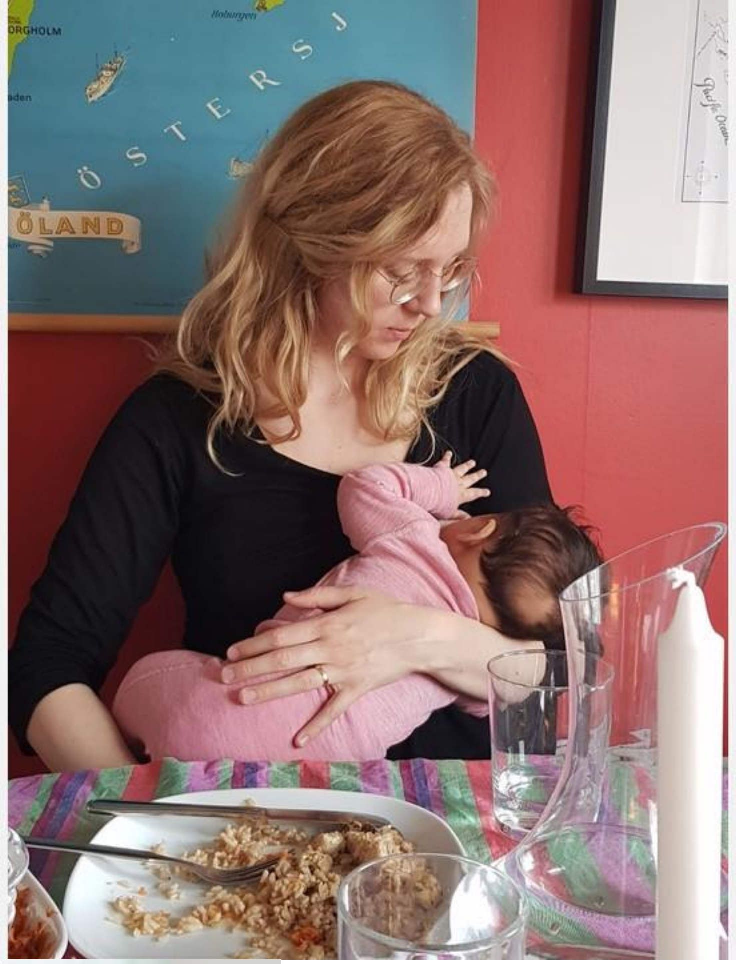

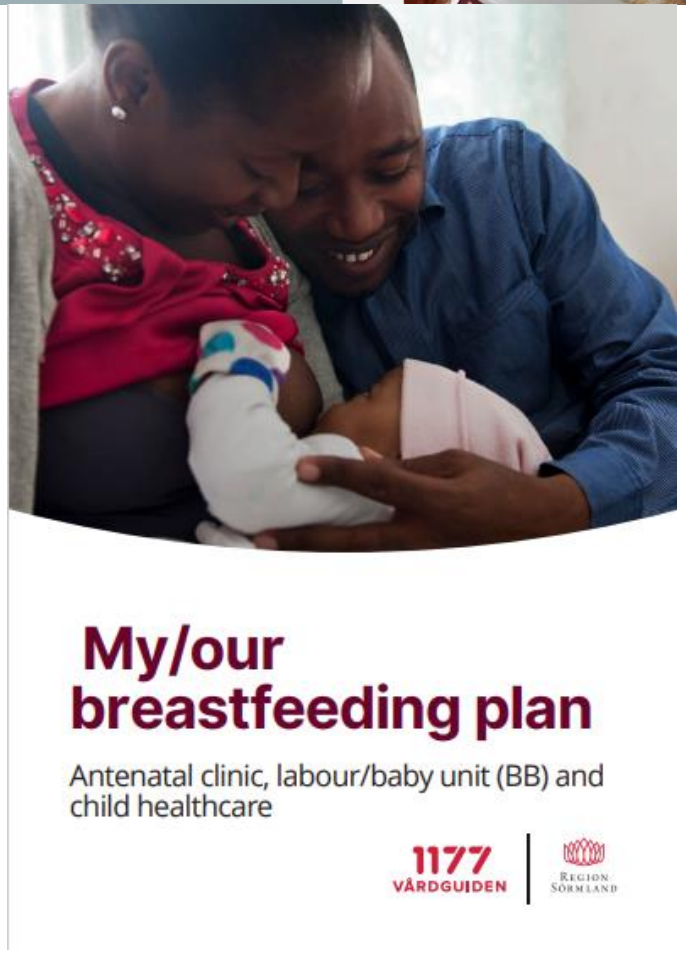

At the antenatal care visit during pregnancy week 32

- Skin-to-skin contact and breastfeeding for the first time
- Go through the breastfeeding plan with parents:

-Pregnant mothers' intentions

-Parents' experiences

-Parents’ expectations

-What kind of breastfeeding support parents want from their family and healthcare professionals

SKIN TO SKIN CONTACT

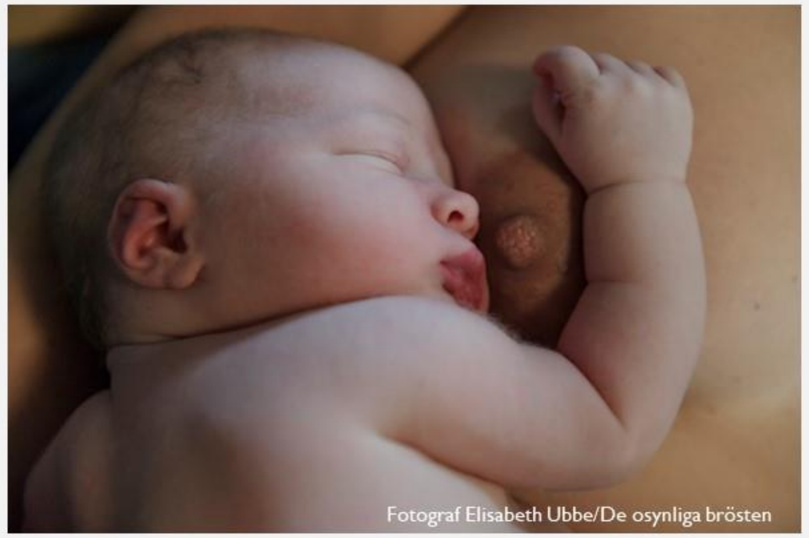

BREASTFEEDING FOR THE FIRST TIME

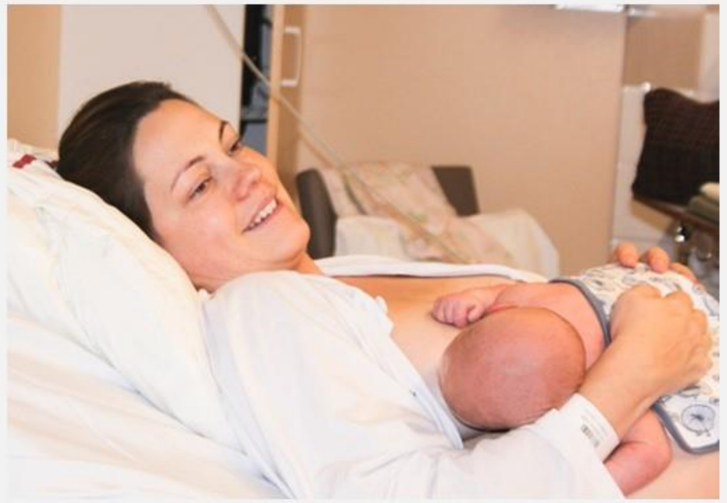

At the antenatal care visit during pregnancy week 38

- How to tell when your baby wants to feed, and that breastfeeding is working

HOW TO TELL YOUR BABY WANTS TO FEED

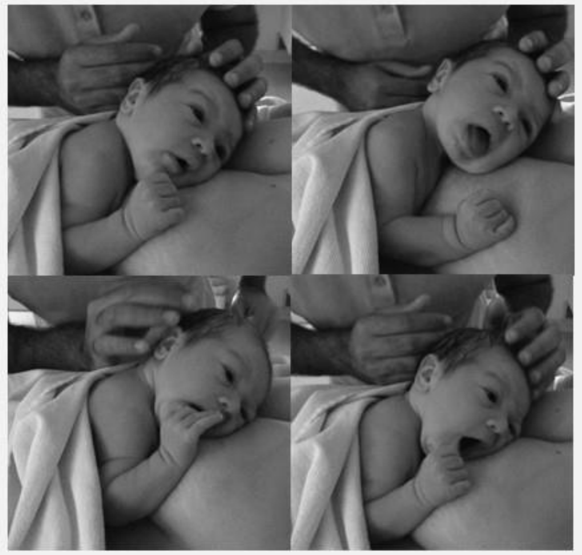

HOW TO TELL THAT BREASTFEEDING IS WORKING

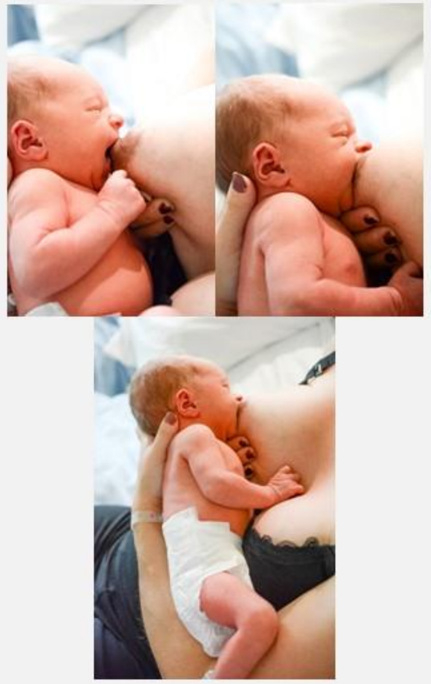

Follow-up after birth, as well as 2 and 8 weeks after birth

- Mothers' intentions, parents' experiences and expectations and what kind of breastfeeding support parents want from their family and healthcare professionals
- If mothers need more breastfeeding support, professionals should help them to contact the breastfeeding outpatient clinic

HOW ARE YOU GETTING ON WITH BREASTFEEDING/FEEDING?

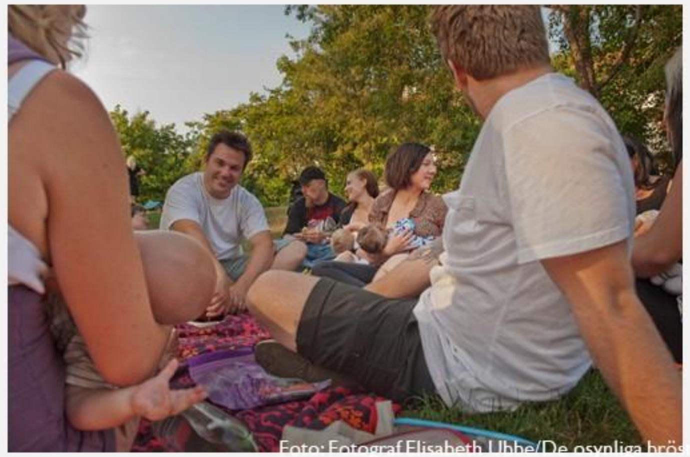

Foto: Fotograf Elisabeth Ubbe/De osynliga brösten

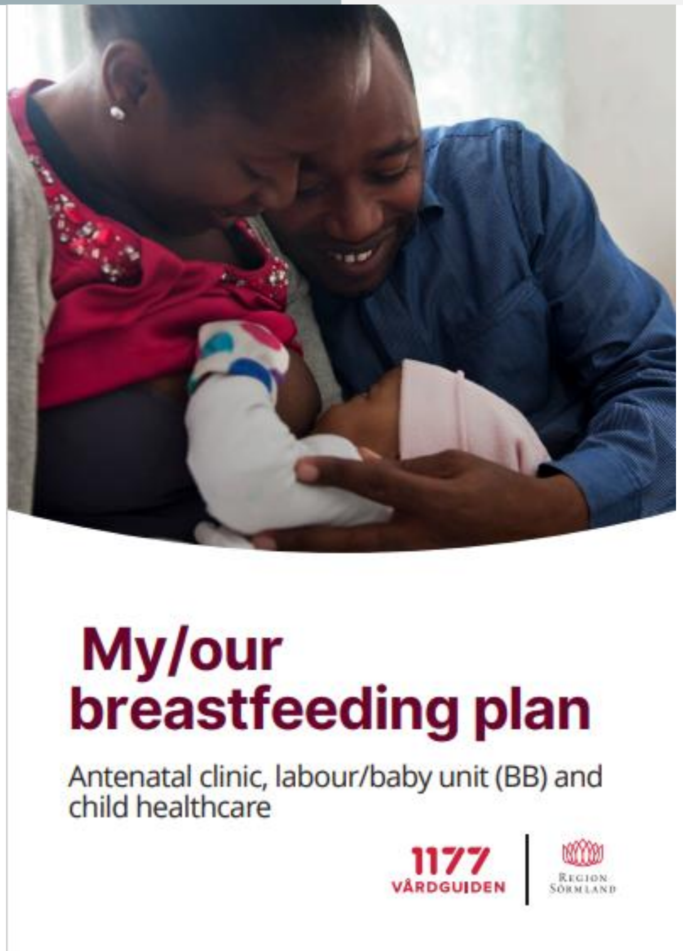

The evidence-based picture and conversation material at the child health centre

TALKING ABOUT CONTINUED CLOSENESS AND BREASTFEEDING IN THE CHILD HEALTH CENTRE

The evidence-based picture and conversation material used by child healthcare nurses at the child health centre: They used empathetic, reflective listening with open-ended questions, reflected and summarised. Healthcare professionals explore what the breastfeeding family already knows about breastfeeding, ask for permission to offer information and offer information.

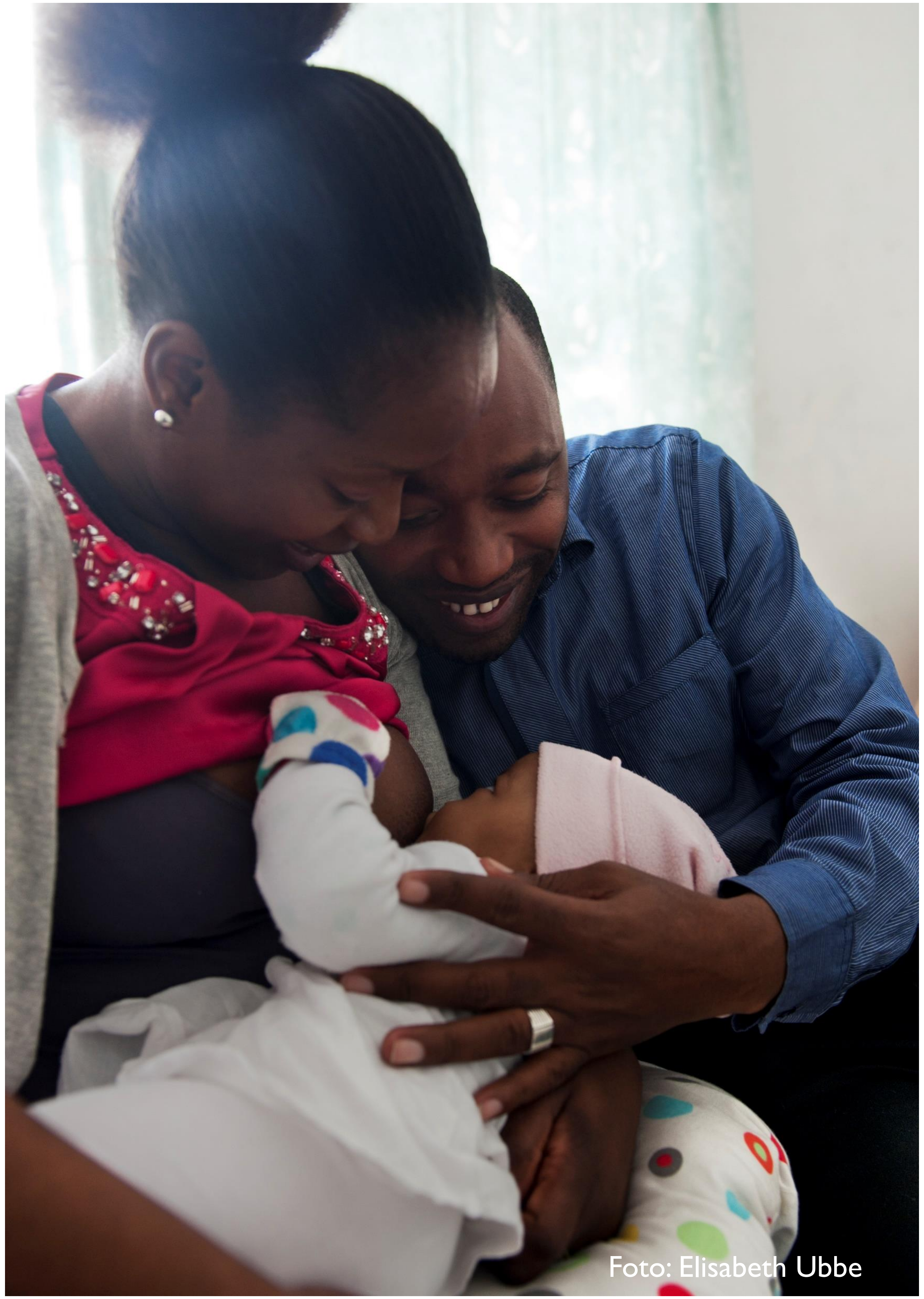

Follow-up at every visit during the breastfeeding period

- Mothers' intentions, parents' experiences and expectations and what kind of breastfeeding support parents want from their family and healthcare professionals
- If mothers need more breastfeeding support, professionals should help them to contact the breastfeeding outpatient clinic

HOW ARE YOU GETTING ON WITH BREASTFEEDING/FEEDING?

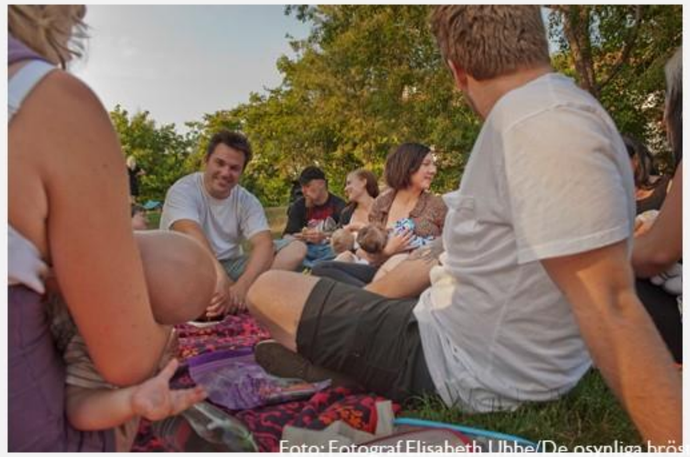

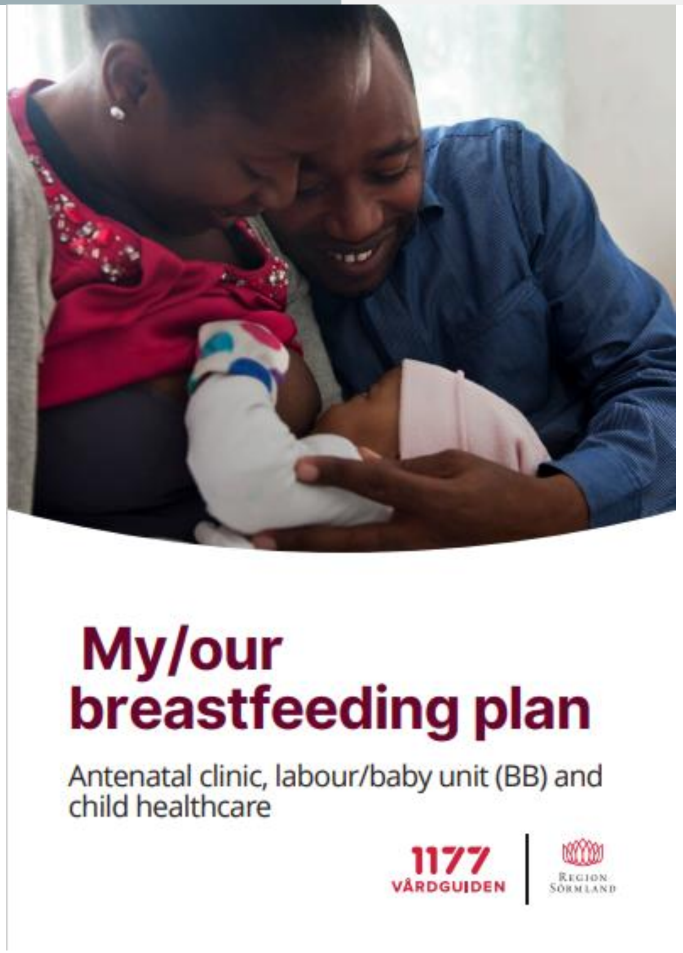

Two to four weeks after birth

- Sleep-deprived

Six to eight weeks after birth

- Development phases

SLEEP-DEPRIVED

The milk production hormone is higher at night

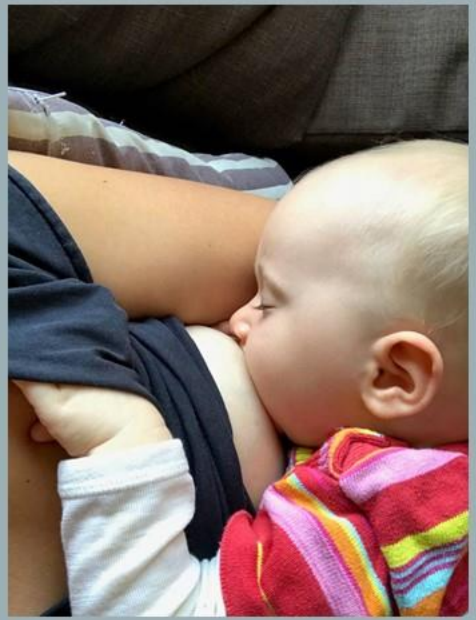

Mothers who breastfeed in the night fall back to sleep faster and sleep more deeply

DEVELOPMENT PHASES

Breastfeeding often and carrying your baby in a sling can help

Your baby will breastfeed more often than before, more times in the night and be fussy at the breast

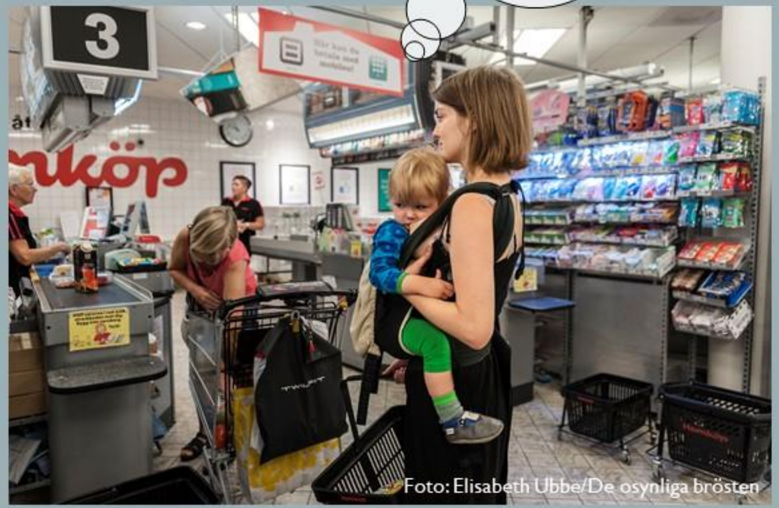

Foto: Elisabeth Ubbe/De osynliga brösten

Breastfeeding plan

Where to get help with breastfeeding during the whole breastfeeding period

Advantages of learning about breastfeeding before the baby is born

Self-studies for parents during pregnancy weeks 28-32: Talk with the partner, family, friends and healthcare professionals about breastfeeding: thoughts, experiences and expectations about breastfeeding

Self-studies for parents during pregnancy weeks 28-32: Talk with the partner, family and healthcare professionals about what kind of support they need from each other, professionals and their family

Self-studies for parents during pregnancy weeks 28-32: Fill in the breastfeeding plan during pregnancy: mothers' intentions about breastfeeding, parents' experiences and expectations and what kind of breastfeeding support parents want from their family and healthcare professionals

Self-studies for parents during pregnancy weeks 32-38: Care routines that can make breastfeeding easier. For example, demand feeding

Self-studies for parents during pregnancy weeks 32-38: Mothers’ own breastfeeding plan about breastfeeding after birth

QR-codes for 4 short online breastfeeding lectures for parents in the following languages: Swedish, English, Somali and Arabic-
Are there any benefits of breastfeeding?
How do you know if breastfeeding is working?
How can the mother experience breastfeeding?
How could the mother continue to breastfeed?

For parents who want to know more about breastfeeding: QR-codes for 2 leaflets, only in Swedish:
Breastfeeding - a good start
Breastfeeding and sleeping well

Fill in the breastfeeding plan before visiting the child health centre during the breastfeeding period:
mothers' intentions about breastfeeding, parents' experiences and expectations and what kind of breastfeeding support parents want from their family and healthcare professionals.

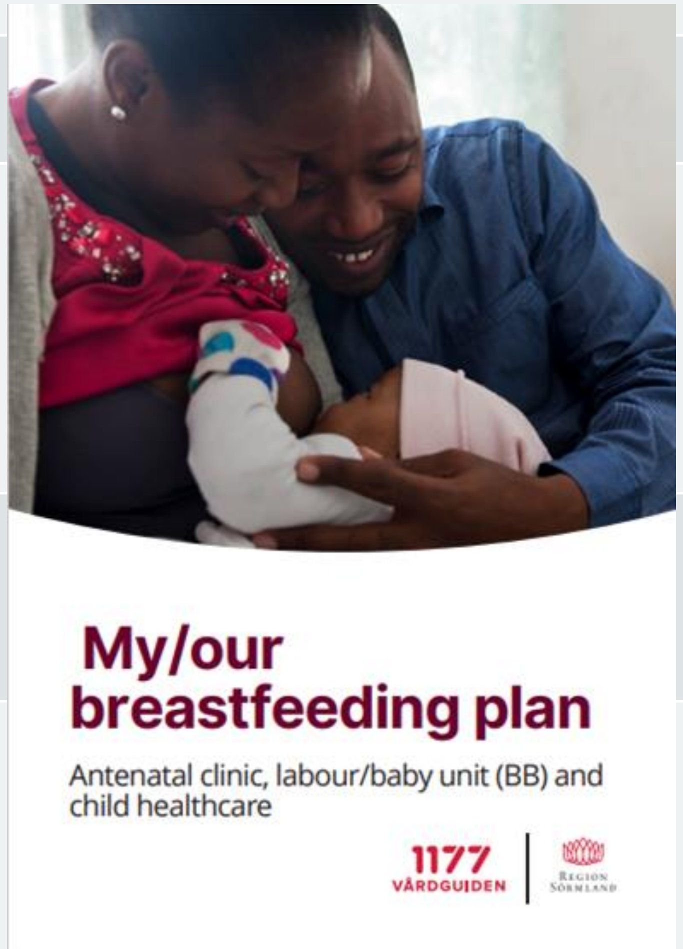

Supplement: Supplementary file 1 — Additional file 1. Supporting information. [file 13006_2023_609_MOESM1_ESM.pdf]
